# Tightly Coupled Integration of GPS Ambiguity Fixed Precise Point Positioning and MEMS-INS through a Troposphere-Constrained Adaptive Kalman Filter

**DOI:** 10.3390/s16071057

**Published:** 2016-07-08

**Authors:** Houzeng Han, Tianhe Xu, Jian Wang

**Affiliations:** 1 School of Environment Science and Spatial Informatics, China University of Mining and Technology (CUMT), Xuzhou 221116, China; hanhouzeng@cumt.edu.cn (H.H.); wjian@cumt.edu.cn (J.W.); 2 State Key Laboratory of Geo-information Engineering, Xi’an 710054, China

**Keywords:** precise point positioning (PPP), inertial navigation system (INS), tightly coupled, ambiguity resolution, troposphere constraint, adaptive filtering

## Abstract

Precise Point Positioning (PPP) makes use of the undifferenced pseudorange and carrier phase measurements with ionospheric-free (IF) combinations to achieve centimeter-level positioning accuracy. Conventionally, the IF ambiguities are estimated as float values. To improve the PPP positioning accuracy and shorten the convergence time, the integer phase clock model with between-satellites single-difference (BSSD) operation is used to recover the integer property. However, the continuity and availability of stand-alone PPP is largely restricted by the observation environment. The positioning performance will be significantly degraded when GPS operates under challenging environments, if less than five satellites are present. A commonly used approach is integrating a low cost inertial sensor to improve the positioning performance and robustness. In this study, a tightly coupled (TC) algorithm is implemented by integrating PPP with inertial navigation system (INS) using an Extended Kalman filter (EKF). The navigation states, inertial sensor errors and GPS error states are estimated together. The troposphere constrained approach, which utilizes external tropospheric delay as virtual observation, is applied to further improve the ambiguity-fixed height positioning accuracy, and an improved adaptive filtering strategy is implemented to improve the covariance modelling considering the realistic noise effect. A field vehicular test with a geodetic GPS receiver and a low cost inertial sensor was conducted to validate the improvement on positioning performance with the proposed approach. The results show that the positioning accuracy has been improved with inertial aiding. Centimeter-level positioning accuracy is achievable during the test, and the PPP/INS TC integration achieves a fast re-convergence after signal outages. For troposphere constrained solutions, a significant improvement for the height component has been obtained. The overall positioning accuracies of the height component are improved by 30.36%, 16.95% and 24.07% for three different convergence times, i.e., 60, 50 and 30 min, respectively. It shows that the ambiguity-fixed horizontal positioning accuracy has been significantly improved. When compared with the conventional PPP solution, it can be seen that position accuracies are improved by 19.51%, 61.11% and 23.53% for the north, east and height components, respectively, after one hour convergence through the troposphere constraint fixed PPP/INS with adaptive covariance model.

## 1. Introduction

Traditionally, the high-precision Global Positioning System (GPS) positioning solution is obtained based on the differential carrier phase measurement. The Real Time Kinematic (RTK) is widely used for high precision applications, which lies in the fact that the related errors between the reference station and rover station can be effectively eliminated or reduced for the short baseline configuration. However, the requirements of deploying a reference receiver will increase the system cost or be problematic. Presently, precise ephemerides and satellite clock products are available from various analysis centers [1,2], such as the International Global Navigation Satellite System (GNSS) Service (IGS). The real time orbits and clocks can be generated by Centre National d’Études Spatiales (CNES) recently [3], also other IGS real time analysis center, e.g., GFZ [4,5]. The precise point positioning (PPP) approach is an efficient positioning technique that uses the undifferenced pseudorange and carrier phase measurements from a single receiver, together with the precise orbit and clock corrections. It can obtain satisfactory positioning performance after convergence [6]. Unnecessary deployment of a reference station makes it a suitable approach for airborne geo-referencing, earthquake or tsunami monitoring applications [7].

Generally, a major limitation of the PPP approach, however, is its requirement of a long convergence time for rapid positioning. The precise results at centimeter-level or millimeter-level are only available after a convergence period. This will inevitably limit its application in dynamic environments. In order to improve the positioning accuracy and reduce the convergence time, the PPP integer ambiguity resolution (IAR) is usually conducted. There are three main available ways to resolve the PPP ambiguities, including the uncalibrated phase delay (UPD) model [4,8], the integer phase clock model [9], and the decoupled clock model [10]. Some researchers clarified that the three approaches are equivalent [11,12], however, the research conducted by Teunissen and Khodabandeh shows that there are differences between the methods [13]. Although significant improvements have been achieved by implementing PPP IAR, a typical convergence time of 20 min is still required for PPP. The GPS IAR performance can actually be improved by integrating GLONASS measurements [14], the convergence time of PPP can be dramatically reduced by combining GPS and GLONASS data for multi-GNSS PPP-AR [15]. In addition, the efficiency of ambiguity resolution in triple-frequency PPP can be significantly improved compared with the dual dual-frequency PPP [16]. On the other hand, by adopting the PPP-RTK strategy, the ambiguities can be resolved instantaneously, thus makes it a suitable approach for real-time applications [17]. However, the deployment of the regional reference network is a prerequisite [18]. The horizontal positioning accuracy can be significantly improved through IAR, while less significant improvement on height components can be achieved, which can be improved by augmenting external tropospheric delay corrections [19]. 

On the other hand, the availability of PPP solutions is seriously restricted by the challenging environments like unban canyons and under foliage. With a complete loss of lock, a re-initialization process will be activated-methods can be found in Geng et al. [20] and Zhang and Li [21]. To overcome these limitations, a commonly used strategy is to include an environment independent system, the Inertial Navigation System (INS), among which the micro-electro-mechanical sensors (MEMS) have gained more attention. Currently, the integrated systems are typically developed based on the integration of differential GPS and inertial sensor for high-precision applications [22,23,24]. In addition, GPS can be integrated with accelerometers for precise dynamic positioning, especially during the seismic events [25,26]. More recently, PPP integrated with INS has been developed for this purpose. The tightly coupled (TC) integration architecture, which has a superior positioning performance and robustness, is commonly applied. Zhang and Gao presented a tightly coupled integration of conventional PPP with INS. Comparable positioning performance has been achieved with the differential GPS/INS integration [27]. Several data sets from airborne environment have been analyzed using PPP/INS integration. The multipass processing has been introduced for post-mission, and centimeter-level positioning accuracy was obtained [28]. The TC integration of conventional PPP and MEMS-based inertial systems using single-differencing operation has been proved to be able to provide decimeter-level positioning accuracy during one hour [29]. The integration of conventional PPP and a tactical inertial measurement unit (IMU) with ionosphere constraint can provide centimeter-level positioning accuracy and improve bridging capability [30]. The positioning performance of fixed PPP/INS integration has not been carefully analyzed yet. In addition, any deficiency in the stochastic models for the GPS measurements will inevitably result in unreliable results, which needs to be carefully considered [31].

In this study, a tightly coupled PPP/INS integration strategy, which combines the IMU and GPS measurements, is introduced. The a priori tropospheric constraint and an adaptive covariance modelling strategy are employed. The PPP IAR is also achievable through integer phase clock model with between-satellites single-difference (BSSD) operation. First, the GPS PPP model is described, focusing on error effects, as well as the PPP IAR method. Then, the PPP/INS tightly coupled model is demonstrated. The inertial sensor biases will be estimated by the integration filter. The tropospheric constrained model and adaptive filtering model are provided subsequently. The field vehicular test and result analysis are presented to evaluate the effectiveness of the proposed algorithm.

## 2. GPS PPP Model

In order to eliminate adverse effects on positioning from the ionospheric delay, the ionospheric-free (IF) combinations of undifferenced dual-frequency GPS pseudorange (*P_IF_*) and carrier phase (Φ*_IF_*) observations are most widely used for PPP [6]. However, the IF ambiguities are estimated as float values in conventional PPP model. In this study, the integer phase clock model is applied to fix the wide-lane (WL) and narrow-lane (NL) ambiguities. The specific method will be described in detail as follows. 

### 2.1. PPP Method

The conventional PPP mathematic model can be written as:

(1) $$P_{IF}=\rho +c\left(dT-dt\right)+T+\varepsilon_{P_{IF}}$$

(2) $$\Phi_{IF}=\rho +c\left(dT-dt\right)+T+\lambda_{IF}N_{IF}+\varepsilon_{\Phi_{IF}}$$

where ρ is the geometric range between satellite and receiver, *c* is the vacuum speed of light, *dT* is the receiver clock error, *dt* is the satellite clock error, *T* is the slant tropospheric delay, λ*_IF_* is the ionospheric-free wavelength (6.3 mm), *N_IF_* is the ionospheric-free ambiguity, $\varepsilon_{P_{IF}}$ and $\varepsilon_{\Phi_{IF}}$ are the relevant pseudorange and carrier phase measurement noise, including the multipath effect.

It can be seen that the first-order ionospheric delay is virtually eliminated though ionospheric-free combinations, and high-order terms are ignored in the model. The satellite clock error and satellite orbit error can be removed by applying the precise clock and ephemerides products provided by the GPS analysis center, the products from CNES were used in our study. The UNB3m tropospheric model [32] combined with Global Mapping Function (GMF) [33] are used to correct the tropospheric hydrostatic delay, and the wet component is estimated as an additional state in the Extended Kalman filter (EKF). Other related error effects (i.e., antenna phase center offsets and variations, differential code bias (DCB), satellite phase wind up, site displacement effects and relativistic effects) are corrected before forming the combinations.

### 2.2. PPP Ambiguity Resolution

In the conventional PPP model, the satellite code clock is applied for both code and carrier phase observations, and the ionospheric-free ambiguities are estimated together with the satellite and receiver code and phase biases, such that the IF ambiguities cannot be fixed to integer values. Practically, it is commonly known that PPP IAR is able to improve the positioning accuracy and reduce the convergence time. As CNES can provide satellite phase clock products, the NL cycles can be recovered to integer values after the correction of measurement with the Grupe de Recherche de Géodésie Spatiale (GRGS) provided satellite phase clock products. The 3D-weighted Root Mean Square (WRMS) of the GRG orbit solutions is around 18 mm with respect to the IGS final products, and the standard deviation of the GRG satellite clock solutions is around 0.12 ns [34]. The accuracy of GRG products enables precise PPP solutions, and PPP IAR is also achievable.

The PPP IAR is then carried out following a two-step procedure. In the first step, the WL ambiguities will be first fixed. The Melbourne-Wübbena combination is commonly used to estimate the WL float ambiguities:

(3) $$\frac{f_{1}\Phi_{1}-f_{2}\Phi_{2}}{f_{1}-f_{2}}-\frac{f_{1}P_{1}+f_{2}P_{2}}{f_{1}+f_{2}}=\lambda_{WL}N_{WL}+b_{WL,r}-b_{WL}^{s}$$

where *f*_1_ and *f*_2_ are the frequency of the GPS signal, Φ*_m_* and *P_m_* are corresponding carrier phase and code observations on frequency band *m*, λ*_WL_* and *N_WL_* are the WL wavelength and ambiguity, *b_WL,r_* and $b_{WL}^{s}$ are WL bias on the receiver and satellite side, respectively.

The WL satellite biases (WSB) term can be corrected using the GRG products. The estimated WL ambiguities are smoothed epoch by epoch to obtain the optimal value. In order to remove the receiver side biases, the BSSD operation is applied, the reference satellite is selected as the highest elevation satellite. The WL IAR is achieved by rounding the float WL ambiguity estimate to the nearest integer value, and the validation decision is made according to the probability-based test [35]. The WL IAR can be easily achieved due to its long wavelength. 

The narrow lane ambiguities (*N_NL_*) are then calculated based on the fixed WL ambiguities and the ionospheric-free ambiguities:

(4) $$\Delta N_{NL}=\frac{\Delta N_{IF}}{17}-\frac{60\Delta N_{WL}}{17}$$

where Δ represents the difference between the base satellite and the other satellites value.

The NL ambiguities are obtained by using the LAMBDA method [36], and ratio test with predefined critical value is carried out to validate the fixed solutions. In this study, the PPP IAR is implemented in an epoch by epoch manner. The fixed NL ambiguities are then used as constrained observations to improve the float solutions.

## 3. Tightly Coupled Integration of PPP and INS

Compared to GNSS, the INS is a self-contained navigation system using the measurements provided by gyroscopes and accelerometers to track the positioning and orientation of a moving object, the INS mechanization is conducted to obtain the navigation solution in the local frame. The major limitation is the unbounded error growth without external corrections, the complementary characteristic of GNSS and INS makes them so well-suited to integration. In this study, the TC integration strategy is implemented using EKF. The TC PPP/INS architecture can provide superior integrity capability and better positioning performance compared to loosely coupled PPP/INS. The TC filter directly fuses IF combined GPS pseudorange, carrier phase observations and INS-derived ranges to estimate the state vector. In the following, the dynamic model and measurement model are developed.

### 3.1. Dynamic Model

The dynamic model is derived from the psi-angle based INS error model [37]:

(5) $$\delta \dot{v}=-(2\omega_{\text{ie}}+\omega_{\text{en}})\times \delta v-\delta \psi \times f+\delta g+\nabla$$

(6) $$\delta \dot{r}=-\omega_{\text{en}}\times \delta r+\delta v$$

(7) $$\delta \dot{\psi }=-(\omega_{\text{ie}}+\omega_{\text{en}})\times \delta \psi +\varepsilon$$

where δr, δv, δψ are the position, velocity and attitude error vectors, respectively; the symbols ∇ and ε are the accelerometer error and gyro error vectors, respectively; δg is the gravity uncertainty error vector, *f* is the specific force vector, ω_ie_ is earth rotation vector, ω_en_ is the craft rate.

A 24 states INS error model is implemented here, which contains nine navigation error states that are expressed in the north-east-down (NED) navigation frame (three for position, three for velocity and three for orientation), six accelerometers (three biases and three scale factors for each axis), three gyro bias errors for each axis, three gravity uncertainty errors and three lever arm errors [22,23]. The detailed error states are given as follows:

(8) $$\left\{ \begin{array}{l} x_{Nav}\;={\left[\delta r_{N},\delta r_{E},\delta r_{D},\delta v_{N},\delta v_{E},\delta v_{D},\delta \psi_{N},\delta \psi_{E},\delta \psi_{D}\right]}^{T}\\ x_{Acc}\;={\left[\nabla_{bx},\nabla_{by},\nabla_{bz},\nabla_{fx},\nabla_{fy},\nabla_{fz}\right]}^{T}\\ x_{Gyro}={\left[\varepsilon_{bx},\varepsilon_{by},\varepsilon_{bz}\right]}^{T}\\ x_{Grav}={\left[\delta g_{N},\delta g_{E},\delta g_{D}\right]}^{T}\\ x_{Ant}\;={\left[\delta L_{bx},\delta L_{by},\delta L_{bz}\right]}^{T} \end{array}\right.$$

The inertial sensor errors are modelled as first-order Gauss-Markov (GM) processes. Additional receiver clock error state, zenith wet tropospheric error state and ionospheric-free ambiguity states are added for the PPP configuration, which can be written as follows:

(9) $$x_{GPS}\;={\left[dT,T,\lambda_{IF}N_{IF}\right]}^{T}$$

In this study, the receiver clock error is modelled as white noise process with a large noise covariance, the tropospheric error is treated as random walk process, and ionospheric-free ambiguities are regarded as random constant.

The corresponding prediction equations can be written as:

(10) $$x_{k+1}^{-}=F_{k}^{k+1}x_{k}$$

(11) $$P_{k+1}^{-}=F_{k}^{k+1}P_{k}F_{k}^{k+1,T}+Q_{k}$$

where $F_{k}^{k+1}$ is the transition matrix from epoch *k* to *k* + 1, $x_{k}$ and $P_{k}$ are the estimated state vector and the corresponding covariance matrix at epoch *k*, $Q_{k}$ is the process noise covariance matrix.

### 3.2. Measurement Model

The measurement model of the TC PPP/INS filter can be expressed as follows:

(12) $$Z_{k}=H_{k}\cdot x_{k}+\varepsilon_{k}$$

where the measurement vector *Z_k_* is defined as the difference between the ionospheric-free pseudorange, carrier phase measurements and the INS predicted measurements, H*_k_* is the design matrix, and $\varepsilon_{k}$ is observation noise vector.

For the conventional stand-alone PPP approach, a considerable time is required to obtain the converged tropospheric solutions [19], considering the correlation between the tropospheric parameter, receiver clock error and height. The precision of other unknown parameters will be degraded during the convergence period. It will be appropriate to make use of external tropospheric corrections to improve the PPP performance. The positioning performance is expected to be improved accounting for the a priori tropospheric constraint, especially for the height component. Practically, the external tropospheric information can be provided by regional reference networks in real-time or near-real time, or the troposphere zenith path delay (ZPD) product from IGS in post-processing mode. In this study, the converged tropospheric zenith total delay (ZTD) estimated from a nearby base station was used as external correction. Actually, the converged values from the rover station can also be used in post-processing mode, due to the slow change characteristic of tropospheric delay. The a priori tropospheric value is updated every half an hour:

(13) $$Z_{trop}=Z_{trop,pre}+\varepsilon_{trop}$$

where the observation noise of the external tropospheric delay can be selected as a loose constraint with an empirical standard deviation.

In addition, an a priori weighting scheme should be used for the GPS observations:

(14) $$\sigma^{2}=R_{r}\left(a^{2}+b^{2}/{\left(\sin\left(E\right)\right)}^{2}\right)$$

where *R_r_* is defined as code/carrier phase error ratio, which is 100 for code observations and 1 for carrier phase observations; *a* and *b* are the carrier phase error factors. The a priori values are selected as 0.003 m; *E* is the satellite elevation angle.

The measurement update equations can be written as follows:

(15) $$K_{k}=P_{k}^{-}H_{k}^{T}{\left(H_{k}P_{k}^{-}H_{k}^{T}+R_{k}\right)}^{-1}$$

(16) $$x_{k}=x_{k}^{-}+K_{k}\left(Z_{k}-H_{k}x_{k}^{-}\right)$$

(17) $$P_{k}=\left(I-K_{k}H_{k}\right)P_{k}^{-}$$

where *K_k_* is the KF gain matrix, *R_k_* is the measurement noise covariance matrix, *I* is the unit matrix.

In order to improve the positioning performance, an optimal stochastic model for GPS measurements can be implemented. For the GPS/INS integration system, the process noise matrix can be determined based on sensor specifications. On the other hand, the Allan variance method can also be used to analyze the stochastic sensor errors. In this study, the process noise is determined with the predefined value, and remains unchanged during the test. The measurement noise covariance matrix is affected by observation environments, and it is more sensitive to the filter performance. In this study, the improved Sage-Huge adaptive algorithm can be written as:

(18) $$R_{k+1}=\left(1-d_{k+1}\right)R_{k}+d_{k+1}\left(v_{k+1}v_{k+1}^{T}+H_{k+1}P_{k+1}{H_{k+1}}^{T}\right)$$

with $d_{k+1}=\left(1-\gamma \right)/\left(1-\gamma^{k+1}\right)$, $v_{k+1}=Z_{k+1}-H_{k+1}x_{k+1}$. Where γ is the forgetting factor, and it is generally in the range between 0.95 and 0.99, $v_{k+1}$ is the residual sequence.

Presently, a considerable time is required for PPP to obtain fixed solutions. As a result, the noise covariance matrix of carrier phase measurements will be updated only when the ambiguities have been fixed to the integer values. By applying the optimal stochastic modelling strategy, the more realistic environment effects (such as multipath effects) can be taken into consideration, thus improved positioning performance is expected for kinematic positioning. 

In order to avoid the harmful effects of abnormal observations or filter failure on the estimation of updated information, a constrained bound is defined:

(19) $$R_{k}\cdot l_{b}\leq v_{k+1}v_{k+1}^{T}+H_{k+1}P_{k+1}{H_{k+1}}^{T}\leq R_{k}\cdot u_{b}$$

where *l_b_* and *u_b_* are the lower bound and upper bound, respectively, which we take as (1/3.5)^2^ and 3.5^2^. The adaptation process can be implemented separately for each code observations, an enlarging factor is also employed.

### 3.3. Integration Implementation

**Figure 1:**
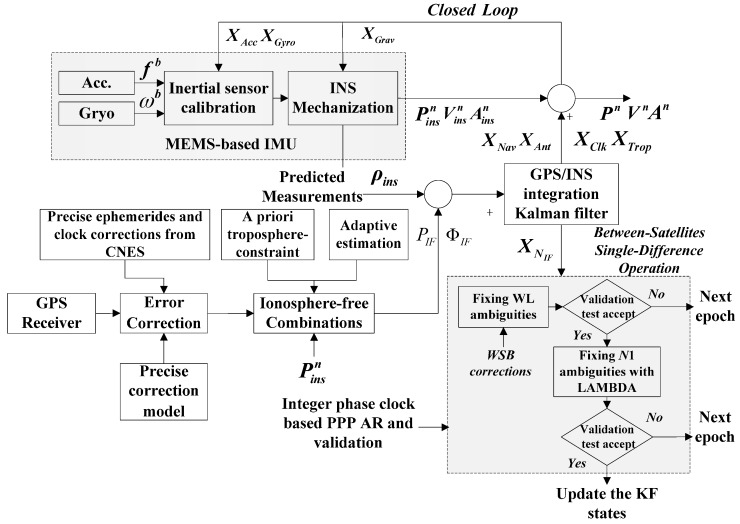
The GPS PPP/INS tightly coupled integration with fixed-ambiguities through a troposphere-constrained adaptive Kalman filter.

In this study, the tightly coupled integration of GPS PPP and INS is implemented by adopting an EKF (Figure 1). The INS initial alignment is first carried out, the INS mechanization is then adopted with compensated sensor outputs given the initial position, velocity and attitude information. The GRG precise ephemerides and clock products are used to correct satellite orbit error and clock error. The integer phase clock can be used for PPP IAR. The tropospheric hydrostatic delay is corrected in advance, and the wet component is estimated as an additional state in the EKF. The other related errors are corrected with the empirical model. The troposphere constrained approach is adopted to improve the positioning accuracy. In addition, an adaptive covariance matrix modelling strategy is employed. The tightly coupled EKF then fuses the undifferenced pseudorange and carrier phase measurements as well as INS predicted ranges with ionospheric-free combinations. A two-step IAR method is activated based on the integer phase clock model. The WL ambiguities are first resolved with WSB corrections after BSSD operation, and afterwards, NL fixing is carried out with fixed WL ambiguities using LAMBDA. The fixed NL ambiguities are then used as constrained observations to update KF states. At last, the navigation solutions from INS are then calibrated with the estimated navigation error states (i.e., the position, velocity, attitude errors and the lever arm offset). The estimated inertial sensor error states are fed back to compensate the raw INS outputs, the gravity uncertainty errors are also fed back to INS mechanization.

## 4. Field Test and Result Analysis

**Figure 2:**
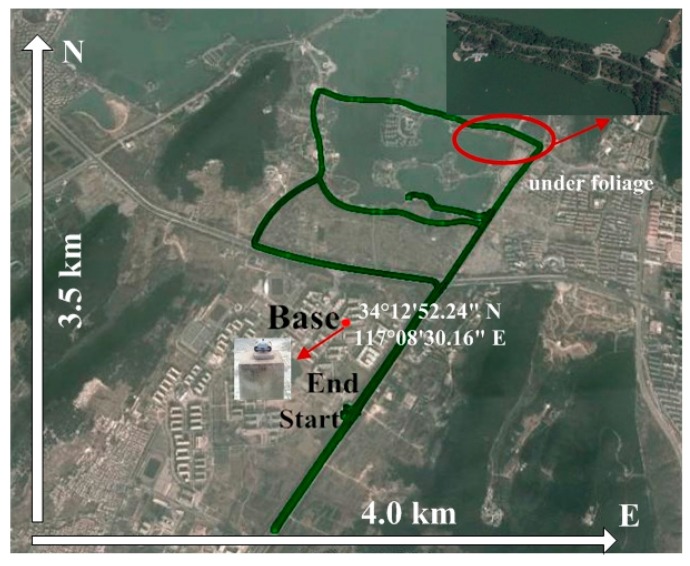
Ground trajectory of vehicular test.

In order to evaluate the performance of the proposed tightly coupled integration algorithm of GPS PPP and INS, a real vehicular navigation test was performed in the district around Yunlong Lake, Xuzhou, China, as described in Han et al. The NovAtel SPAN-CPT system, including fiber optic gyros with the bias stability of ±1°/h and MEMS accelerometers with the bias stability ±0.75 mg, and one high-grade geodetic GNSS dual-frequency receiver were used to collect data. The SPAN-CPT was operated in single positioning mode in real time, thus several meters level positioning accuracy can be obtained, and the obtained solution cannot used as reference. A total of about 70 min data were collected during our trial. The raw GPS data were recorded at a 1 Hz rate, and the raw IMU data were collected at a rate of 100 Hz. The test trajectory is shown in Figure 2. The integrated system operated under an open sky condition for most of the time.

In order to provide a reference solution, a nearby base station was set up on the roof of the School of Environment Science and Spatial Informatics (SESSI) building (Base), on the campus of China University of Mining and Technology (CUMT). The base station operated under a favorable environment, which was seldom affected by the multipath disturbance. The rover-base separation is less than 5 km and mean rover-base height difference is around 25 m during the test, such that the atmospheric delay between the base station and rover station can be neglected. The tropospheric parameters derived from the based station can be assumed accurate enough as constraint. Specifically, the accuracy of external corrections is independent of vehicle dynamics. 

In our processing scheme, the position of the base station was calculated using PPP strategy with daily observations by ambiguity fixing. The reference solution was obtained from the carrier phase based differential GPS/BeiDou/INS tightly coupled system, and the backward smoothing was also carried out. The obtained reference position is at the level of 1–2 cm, thus providing superior positioning accuracy compared to the schemes used in this study. The accuracies of velocity and attitude of the GPS/BDS RTK/INS integrated system stay at the level of 1–2 cm/s and 0.03° in the post-processing mode.

**Figure 3:**
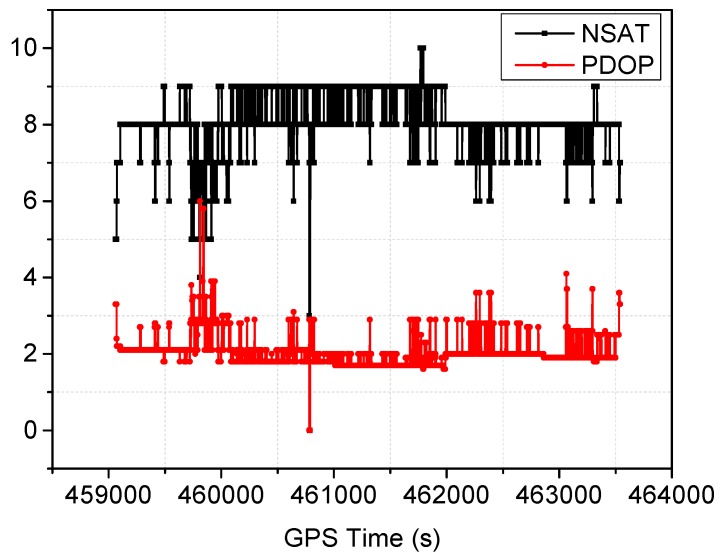
The number of visible satellites and PDOP during the test.

The satellite visibility and PDOP variations during the test can be seen in Figure 3. The average number of visible satellites is 8.1, and the mean PDOP is 2.1. The elevation mask angle was set as 10°. The stand-alone PPP can work well for most of the time when the number of visible satellites is not less than five. If less than five satellites are observed currently, the PPP solutions become unstable. During the test, a 6 s (GPS Time: 460,783–460,788 s) complete signal loss of lock occurred when the vehicle was driving under foliage, which is highlighted by a red circle in Figure 2. During the outage period, the PPP solutions become unavailable; then the PPP requires a re-initialization. However, with a tightly coupled PPP and INS system, the solution can still be available using GPS measurements when less than five satellites are observed, and precise INS-alone solutions are obtainable for short complete signal outages. In order to evaluate the positioning performance of the integrated system. Six different data processing schemes are designed:

Scheme 1—Conventional PPP;
Scheme 2—Conventional PPP/INS;
Scheme 3—Fixed PPP/INS;
Scheme 4—Troposphere-constraint fixed PPP/INS;
Scheme 5—Troposphere-constraint conventional PPP/INS with adaptive R;
Scheme 6—Troposphere-constraint fixed PPP/INS with adaptive R.

The INS was initialized with coarse alignment and fine alignment, the start values for position and velocity are obtained from the RTK solutions, however a large initial standard deviation was applied, 0.15 m for position and 0.1 m/s for velocity, respectively. Actually, the PPP alone usually performs more stable, however, in order to improve the convergence rate, the PPP IAR was conducted using integer phase clock model, which is beneficial to dynamic navigation applications with limited data span. On the other hand, the incorrect fixed ambiguities result in degraded performance, thus, relative strict AR thresholds were applied in this research.

The probability threshold of WL fixing decision is set as 0.9999, the fractional part of the float WL ambiguities larger than 0.25 cycles are also excluded, the NL ambiguities are fixed using LAMBDA after validation with the critical value of 3.0. The quality control strategy should also be applied. The TC strategy is implemented in this study, the process noise covariance matrix is determined based on IMU sensor specifications. The a priori tropospheric zenith total delay value is 2.446 m and stays unchanged, the corresponding standard deviation is 0.03 m.

**Figure 4:**
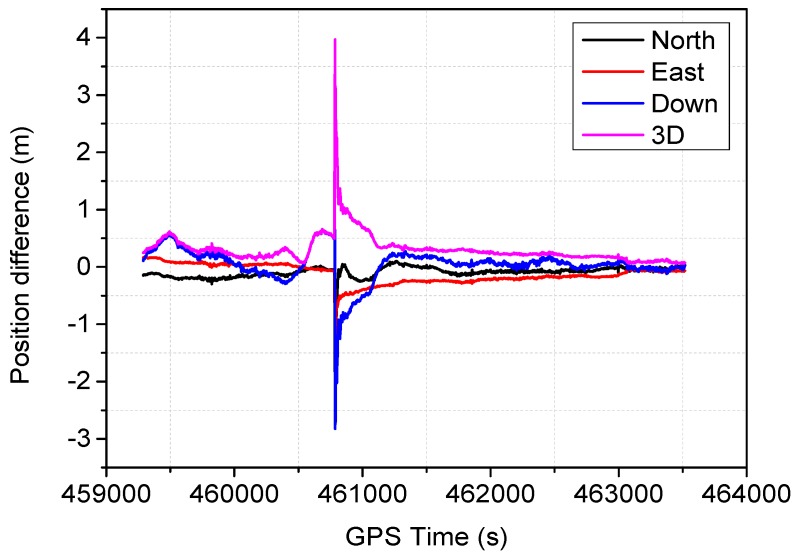
Position difference from the conventional PPP.

**Figure 5:**
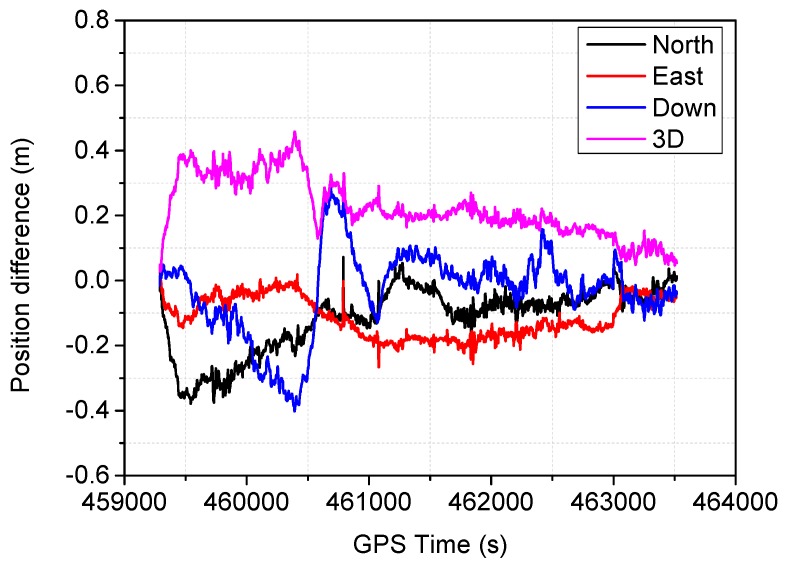
Position difference from the fixed PPP/INS.

**Figure 6:**
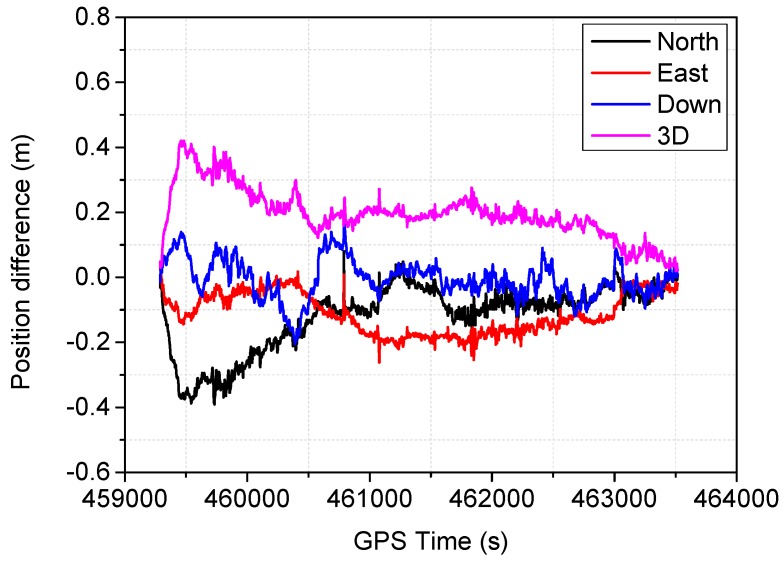
Position difference from the troposphere-constraint fixed PPP/INS.

**Figure 7:**
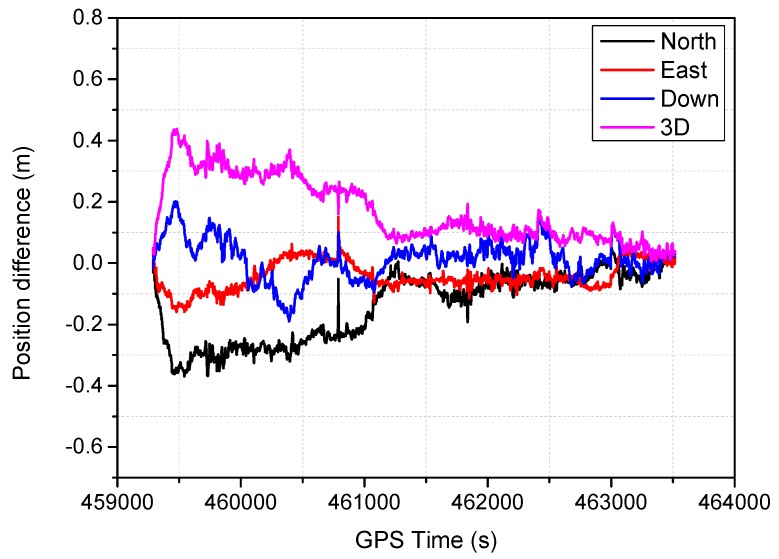
Position difference from the troposphere-constraint fixed PPP/INS with adaptive R.

The positioning difference with respect to the reference solution using schemes 1, 3, 4 and 6 are shown in Figure 4, Figure 5, Figure 6 and Figure 7, similar positioning results for schemes 2 and 5 are not shown. Decimeter-level positioning accuracy is achievable for the whole test trajectory with INS aiding. The positioning accuracy has been improved after the convergence period. Favorable positioning results can be obtained when the GPS signal availability is good. However, it can be seen in Figure 4, a longer time is required to converge for the stand-alone PPP. When the vehicle was driving under foliage, the complete signal loss of lock occurred. A re-initialization process is required and the positioning accuracy is obviously degraded for GPS alone. It can also be seen that a slower convergence rate occurs for the east component. With the inertial aiding, the positioning performance has been improved, especially for the east component (Figure 5). A significant advantage can be seen during the GPS outage by making use of the inertial information, it has been shown the positioning accuracy has not been significantly degraded, which significantly shortens the re-initialization time. In this dynamic test, the observation noise is changed due to varied environments. In order to avoid wrong fixing of ambiguities, a strict threshold of fixing decision was set, and a relative low fixing rate can be obtained for the test data. Such that, no significant difference on positioning accuracy can be seen for the fixed PPP/INS integration compared to the conventional PPP/INS integration. By constraining the tropospheric delay, the height component of the fixed solutions has been obviously improved (Figure 6). This is due to the tropospheric constraint approach can efficiently de-correlate among the tropospheric delay and height component. By applying adaptive procedure, the covariance matrix of measurement noise is adapted based on the filtering residuals, which results in a better positioning performance, especially for the horizontal component (Figure 7). It takes about 220 s for troposphere constraint fixed PPP/INS with adaptive covariance to converge at the level of 0.2 m in 3D position after the outage period occurs, which indicates a fast convergence rate.

**Figure 8:**
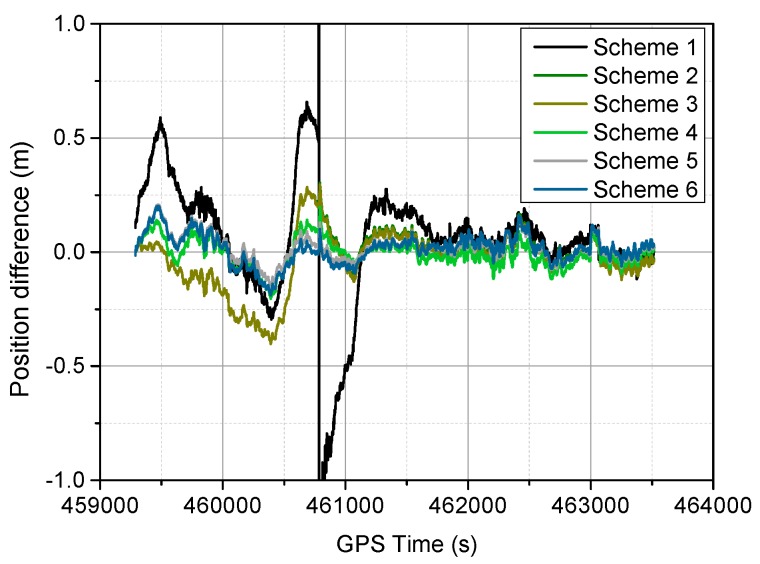
Height position difference for different schemes.

**Figure 9:**
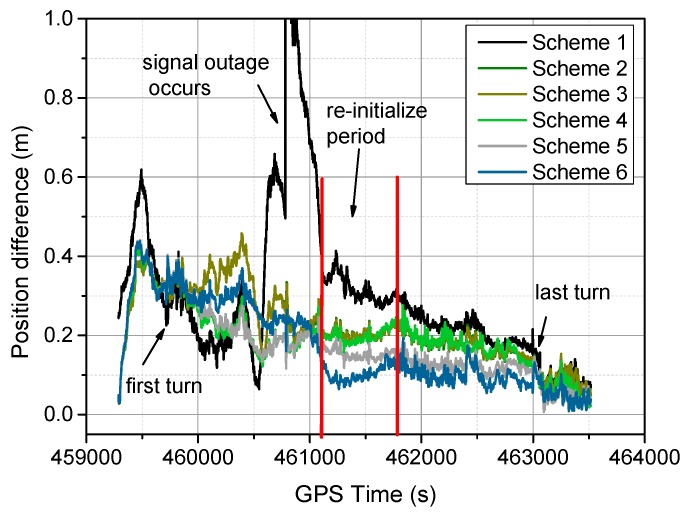
3D position difference for different schemes.

**Figure 10:**
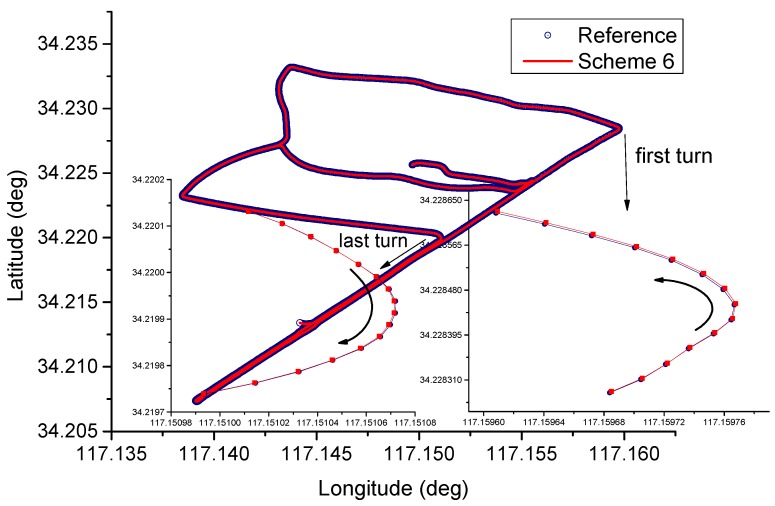
Vehicle trajectories comparison.

In Figure 8, the height position differences for different schemes are shown, and the corresponding 3D position differences are shown in Figure 9, the signal outage period and filter re-initialization period are also shown in Figure 9. It can be seen the troposphere constrained scheme improves the height accuracy, and the troposphere constraint fixed PPP/INS with adaptive filtering achieves faster re-convergence rate and better positioning performance. In Figure 10, the vehicle ground trajectories are shown. It can be seen that the obtained solution using scheme 6 is closely consistent with the reference solution, slight deviation occurs during the vehicle turning period, which can also be seen in Figure 9.

**Figure 11:**
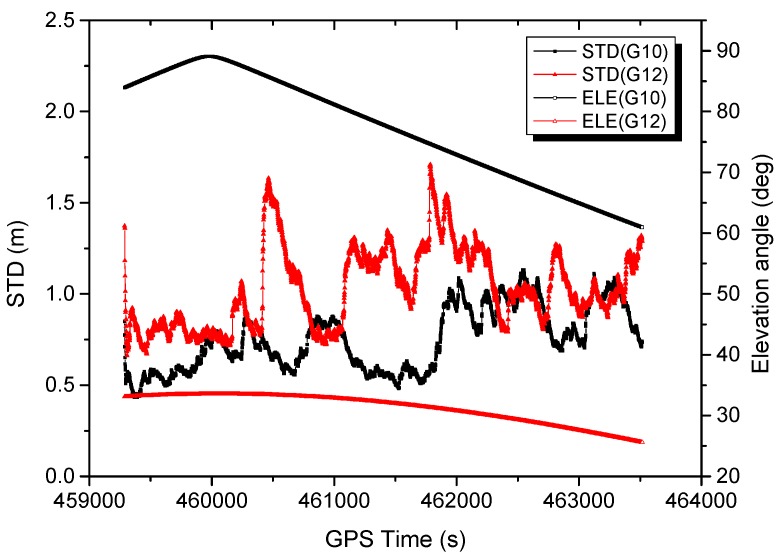
Estimated IF code standard deviation using adaptive strategy.

In Figure 11, the estimated IF code standard deviations (STD) using the adaptive strategy with scheme 6 are presented. It can be seen that the code STD can be adapted on-line considering the environment changes with time. The measurements obtained from different satellites have varying noise levels, the measurement at low elevation angles are generally nosier.

In order to evaluate the positioning performance, the root mean square (RMS) of the difference in position derived from the deployed strategy compared with the reference solution has been calculated. As we known, PPP performance is closely related to convergence time. Conventional PPP usually requires a convergence time longer than 30 min to achieve desired positioning accuracy of 10 cm. In addition, frequent signal outages may need a re-initialization process, and the initialization and re-initialization are the major limitation on PPP for rapid and real-time positioning. In our processing schemes, the data span of our trial is about 70 min, three different evaluation sessions are designed based on the time span of the convergence period, that is, the RMSs are calculated using the time series of position difference from the starting epoch at 30, 50 and 60 min, respectively, to the end epoch of the data set. By using three different convergence periods, the convergence performance can be accurately evaluated. Statistical analysis of the positioning difference is provided in Table 1. Comparing the RMSs of each solutions, it can be seen that position solutions reach centimeter-level accuracy for all models after one hour convergence time. When aiding with inertial sensor measurements, the positioning performance is improved and convergence time is shortened. With the a priori tropospheric constraint, the overall position accuracies of the height component for three sessions are improved from 5.6/5.9/5.4 to 3.9/4.9/4.1 cm. Corresponding to an improvement of 30.36%, 16.95% and 24.07% for the three sessions, a slight degradation for the improvement in the second session is mainly caused by the reducing of satellite availability occurred in the session (Figure 3), thus results in worse observation geometry. By applying the adaptive filtering procedure, the positioning performance has been improved. The ambiguity resolved solutions achieve obvious improvements for the east component. The accuracies are improved from 5.0/8.0/10.8 to 3.5/4.8/5.5 cm, which indicate improvements of 30.00%, 40.00% and 49.07% for the three sessions, respectively. However, a slight degradation occurs for the north component. When compared with the conventional PPP solution, it can be seen that position accuracies are improved by 19.51%, 61.11% and 23.53% for the north, east and height components, respectively, after one hour convergence through the troposphere constraint fixed PPP/INS with adaptive covariance model. It also indicates that height component has a faster convergence rate using the proposed model. Overall, the PPP positioning performance in the dynamic trail is evidently worse than that in static conditions, which is due to more challenging environments encountered, such as, multipath effects, satellite visibility and vehicle dynamics. In addition, the data length for dynamic trail is limited.

**Figure 12:**
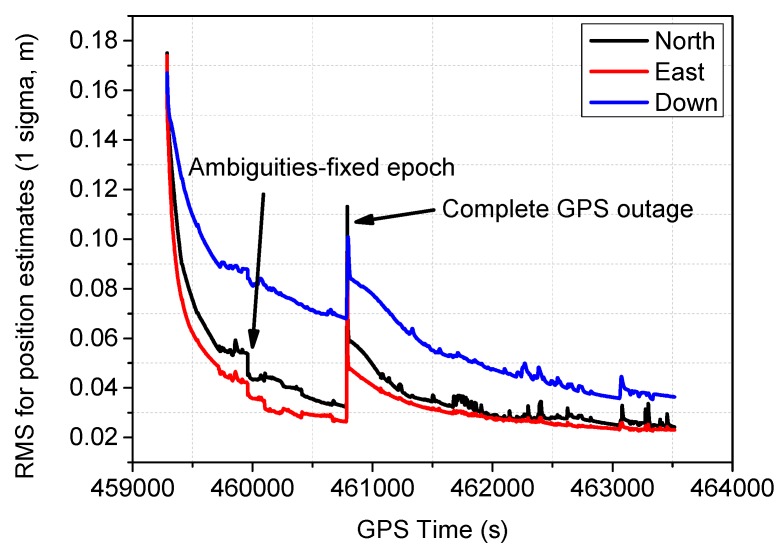
RMS for position estimates derived from the adaptive Kalman filter.

The estimated RMSs for position derived from the diagonal elements of KF using scheme 6 are shown in Figure 12, this figure indicates centimeter-level accuracy can be obtained for the position component after long convergence time, it also shows that the RMSs can be obviously decreased by fixing the ambiguities, and inevitably increasing for the RMS can be observed when the signal outage occurs. 

**Figure 13:**
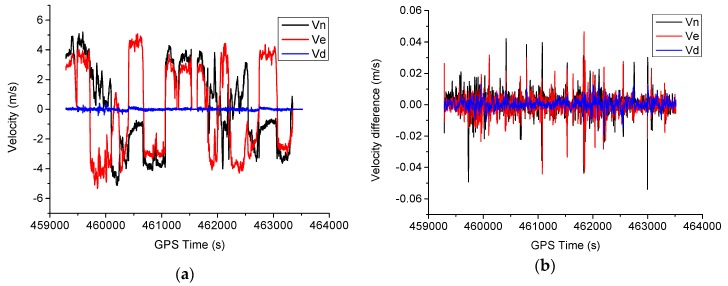
Velocity estimates and difference for the test. (**a**) Velocity estimates; (**b**) Velocity difference.

The velocity estimates using scheme 6 during the test are shown in Figure 13a, and the corresponding velocity differences are shown in Figure 13b. It can be seen that no convergence time is required for the velocity estimates, and dynamics are mainly observed in the horizontal direction. The RMS of the velocity difference are 0.7, 0.6 and 0.3 cm/s for north, east and height components, respectively, and the velocity accuracy of the height component is slightly better than that of horizontal components. The large velocity error usually occurs at the epoch when the vehicle dynamics change significantly. It has been found that no significant difference exists using different models aided with INS.

**Figure 14:**
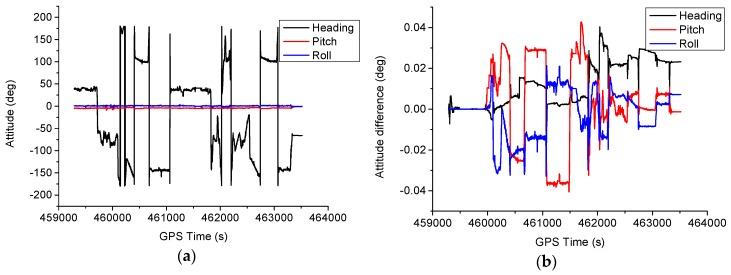
Attitude estimates and difference for the test. (**a**) Attitude estimates; (**b**) Attitude difference.

The attitude estimates using scheme 6 are shown in Figure 14a. The corresponding attitude differences are shown in Figure 14b. It can be seen that the attitude variations mainly occurs for heading component. The RMS of the attitude difference are 0.017, 0.019 and 0.012 degrees for heading, pitch and roll components, respectively.

**Figure 15:**
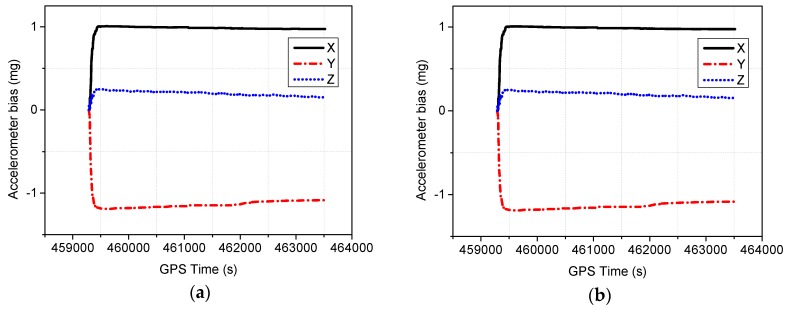
Estimated accelerometer bias and gyroscope bias for the test. (**a**) Accelerometer bias; (**b**) Gyroscope bias.

In the TC integration strategy, the inertial sensor errors are estimated as additional parameters. The estimated sensor biases are then fed back to compensate the raw IMU outputs. Figure 15a shows the estimated accelerometer bias, and the corresponding gyroscope bias estimates are shown in Figure 15b. As can be seen in the figures, the estimated sensor biases become stable after the initial convergence period, and the estimated values can meet the IMU sensor specifications.

## 5. Conclusions 

In this study, the TC integration of PPP and INS is implemented through an EKF. The dynamic model and PPP measurement model are demonstrated. The PPP IAR is conducted based on the integer phase clock model with BSSD operation. The a priori troposphere constrained approach is utilized to improve the positioning performance, and an adaptive filtering strategy is applied to construct an optimal covariance matrix. The proposed algorithm was validated using a field vehicular test. 

The results confirm that integrated system can achieve centimeter-level positioning accuracy, and superior positioning performance and convergence performance have been obtained with INS aiding compared to the stand-alone PPP approach. The integrated system can easily recover from short signal outages. It has been found that height accuracy is significantly improved with the a priori tropospheric constraint. The accuracies are improved by 30.36%, 16.95% and 24.07% (from 5.6/5.9/5.4 to 3.9/4.9/4.1 cm) for three different convergence times, i.e., 60, 50 and 30 min, respectively, while less significant improvements have been found for the horizontal components. By conducting PPP IAR, obvious improvement can be found for horizontal components, especially for the east component. When compared with the conventional PPP solution, it can be seen that the position accuracies are improved by 19.51%, 61.11% and 23.53% (from 5.0/8.0/10.8 to 3.5/4.8/5.5 cm) for the north, east and height components, respectively, after one hour convergence through the troposphere constraint fixed PPP/INS with adaptive covariance model.

In addition, the results have confirmed that the several mm/s accuracy in terms of RMS for velocity estimates can be achieved for the PPP/INS integrated system, and a slightly better accuracy has been obtained for the height component. The attitude difference with respect to the reference solution obtains an accuracy better 0.02° in terms of RMS.

In this study, the IAR is carried out in the PPP/INS TC integration system, however, the IAR performance will be limited due to the challenging environments in the dynamic test, and the future study will be concentrated on improving the IAR performance based on multi-GNSS.

## Figures and Tables

**Table 1:** RMS of the position difference from six different schemes with respect to the reference solution.

Scheme	After 60 min (cm)			After 50 min (cm)			After 30 min (cm)		
	North	East	Down	North	East	Down	North	East	Down
1	4.1	9.0	5.1	5.8	13.6	6.7	7.1	20.2	10.1
2	3.8	7.0	5.5	5.3	11.1	5.9	6.3	15.0	5.7
3	3.8	7.1	5.6	5.3	11.1	5.9	6.3	15.0	5.4
4	4.4	6.0	3.9	6.2	10.6	4.9	7.1	14.9	4.1
5	3.1	5.0	3.9	3.9	8.0	4.8	4.9	10.8	4.9
6	3.3	3.5	3.9	4.5	4.8	5.0	6.5	5.5	4.4
